# Rusty Microglia: Trainers of Innate Immunity in Alzheimer's Disease

**DOI:** 10.3389/fneur.2018.01062

**Published:** 2018-12-04

**Authors:** Adonis Sfera, Roberto Gradini, Michael Cummings, Eddie Diaz, Amy I. Price, Carolina Osorio

**Affiliations:** ^1^Psychiatry, Loma Linda University, Loma Linda, CA, United States; ^2^Patton State Hospital, San Bernardino, CA, United States; ^3^Department of Pathology, La Sapienza University of Rome, Rome, Italy; ^4^Evidence Based Medicine, University of Oxford, Oxford, United Kingdom

**Keywords:** inflammasomes, astrocytes, microglia, exosomes, trained immunity, tolerized immunity

## Abstract

Alzheimer's disease, the most common form of dementia, is marked by progressive cognitive and functional impairment believed to reflect synaptic and neuronal loss. Recent preclinical data suggests that lipopolysaccharide (LPS)-activated microglia may contribute to the elimination of viable neurons and synapses by promoting a neurotoxic astrocytic phenotype, defined as A1. The innate immune cells, including microglia and astrocytes, can either facilitate or inhibit neuroinflammation in response to peripherally applied inflammatory stimuli, such as LPS. Depending on previous antigen encounters, these cells can assume activated (trained) or silenced (tolerized) phenotypes, augmenting or lowering inflammation. Iron, reactive oxygen species (ROS), and LPS, the cell wall component of gram-negative bacteria, are microglial activators, but only the latter can trigger immune tolerization. In Alzheimer's disease, tolerization may be impaired as elevated LPS levels, reported in this condition, fail to lower neuroinflammation. Iron is closely linked to immunity as it plays a key role in immune cells proliferation and maturation, but it is also indispensable to pathogens and malignancies which compete for its capture. Danger signals, including LPS, induce intracellular iron sequestration in innate immune cells to withhold it from pathogens. However, excess cytosolic iron increases the risk of inflammasomes' activation, microglial training and neuroinflammation. Moreover, it was suggested that free iron can awaken the dormant central nervous system (CNS) LPS-shedding microbes, engendering prolonged neuroinflammation that may override immune tolerization, triggering autoimmunity. In this review, we focus on iron-related innate immune pathology in Alzheimer's disease and discuss potential immunotherapeutic agents for microglial de-escalation along with possible delivery vehicles for these compounds.

## Highlights

- LPS activates microglial TLRs, training these cells excessively and triggering the release of C1q, TNF-alpha, and IL-1 alpha, biomolecules associated with autoimmunity.
- A1 astrocytes, likely autoimmune, eliminate viable neurons and oligodendrocytes, contributing to Alzheimer's disease neuronal and synaptic loss.
- LPS, a danger signal, promotes intracellular iron sequestration, increasing the risk of ROS. LPS also promotes ferritinophagy, increasing the free intracellular iron levels.
- ROS and iron activate NLRP3 inflammasomes, generating prolonged inflammation which may override microglial tolerization, engendering autoimmune A1 astrocytes.
- Promoting tolerization and lowering training may de-escalate microglia, lowering the risk of neuronal loss and Alzheimer's disease.

## Introduction

Alzheimer's disease (AD), the most common form of dementia, is marked by progressive memory impairment and functional decline believed to reflect synaptic and neuronal loss. Elimination of these structures may be carried out by an aggressive astrocytic phenotype, defined as A1. Preclinical studies show that LPS-activated microglia enable the conversion of trophic to A1 astrocytes by releasing several cytokines, including tumor necrosis factor alpha (TNF-alpha), interlukin-1 alpha (IL-1 alpha), and complement component C1q. These molecules alter astrocytic transcriptomes, promoting the neurotoxic A1 cells observed to engage in the phagocytosis of healthy neurons and oligodendrocytes, contributing to their loss (1). In this article we take the position that A1 astrocytes are autoimmune in nature, resulting from the prolonged microglial activation that overrides LPS tolerization.

LPS tolerization refers to the absence of an inflammatory response after repeated or prolonged exposure to this microbial endotoxin as re-challenged “tolerant” innate immune cells are incapable of immunological activation. This is relevant to AD because the constantly elevated brain LPS levels, detected in this disorder, fail to trigger microglial tolerization and lower neuroinflammation (2, 3).

**Figure 1:**
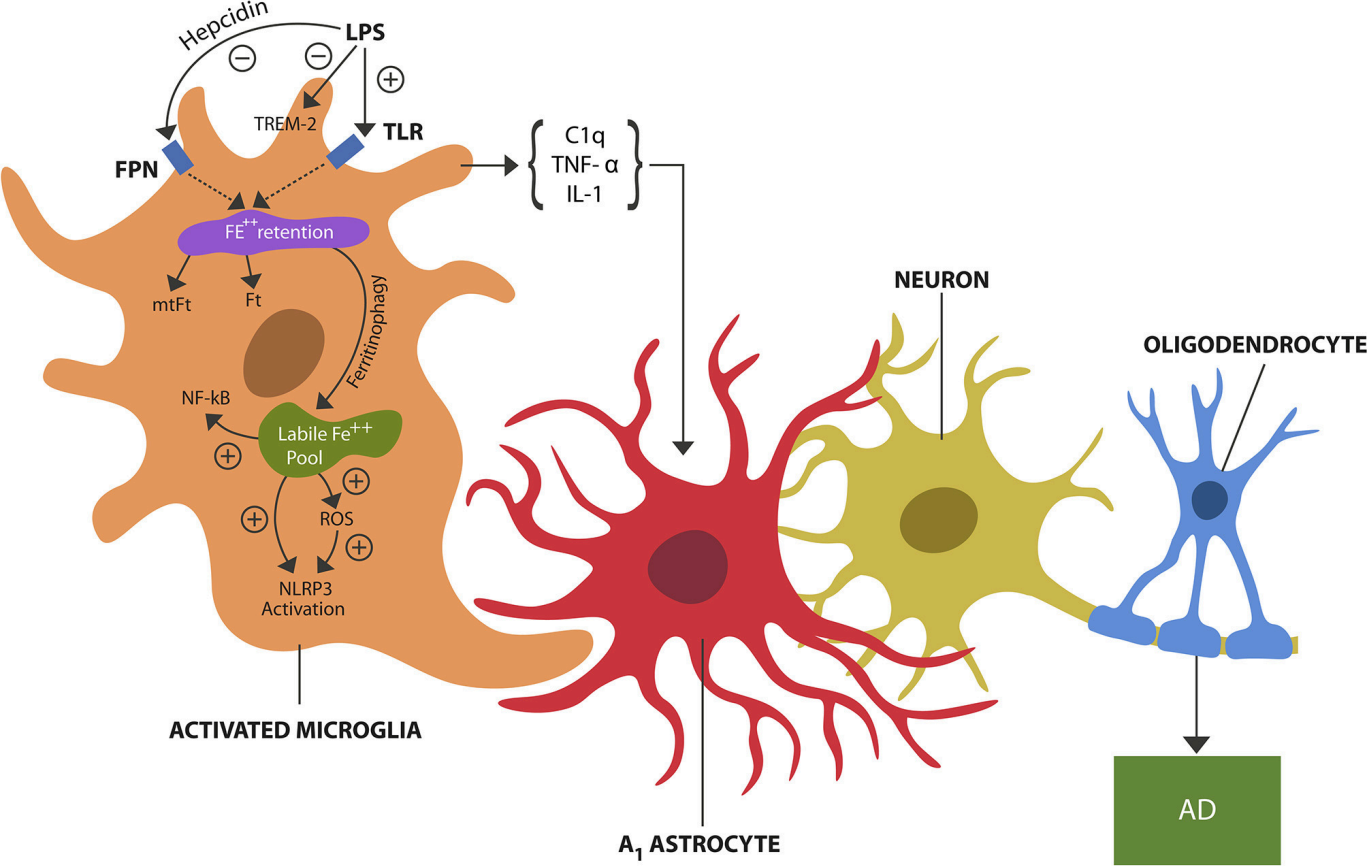
LPS, a danger signal, binds to TLRs of microglia, inducing pathogen-mediated intracellular iron sequestration. LPS also promotes astrocytic hepcidin synthesis which via FPN prevents iron egress. In addition, LPS inhibits TREM-2 expressed on microglial cells. Intracellular iron is stored in Ft and mtFt. Iron can dissociate from ferritins via ferritinophagy, augmenting the free iron (labile iron pool). Unbound iron directly and indirectly (via ROS) activates NLRP3 and NF-kB, inducing microglial activation (training). Microglial release of cytokines, including TNF-alpha, IL-1, and C1q induces A1 astrocytes shown to engage in the phagocytosis of viable neurons and oligodendrocytes, contributing to neuronal loss and possibly AD.

Excess LPS in the brain microenvironment, a danger signal, is detected by microglial TLRs which activate the pathogen-mediated iron sequestration via hepcidin-ferroportin axis (4) (Figure 1). Indeed, recent neuroimaging studies detected iron-containing microglia in the hippocampi of AD patients, likely reflecting LPS-triggered iron sequestration (5).

The source of brain LPS is unclear at this time, but it has been hypothesized that it may derive from the gastrointestinal (GI) tract microbial community. These bacteria were found to lie dormant in various tissues, possibly including the brain, for prolonged periods of time restrained by the lack of free iron (3, 6). Later in life, impaired iron homeostasis caused by aging and/or defective ferritinophagy may provide these microbes with enough metal to resume their growth and LPS shedding, engendering inflammatory neuropathology (6).

Iron is closely linked to immunity as it plays a key role in immune cells proliferation and maturation, but it is also indispensable to pathogens and malignancies which compete for its capture (discussed in The Trained Arm of Innate Immunity section). To safeguard iron, innate immune cells, including microglia and astrocytes, sequestrate it intracellularly bound to cytosolic and mitochondrial ferritin, an arrangement which can generate excessive ROS and inflammation when disrupted (4). Indeed, iron-ferritin dissociation was demonstrated in AD animal models in which LPS-induced ferritinophagy increased intracellular free iron, engendering neuroinflammation (7, 8). Moreover, iron-damaged mitochondria were shown to act as danger signals, activating the inflammatory cascade by igniting inflammasomes, including NLRP3 (9).

LPS-activated microglia release cytokines, including TNF-alpha, IL-1 alpha, and C1q, that have been associated with autoimmune inflammation, an association that led to the development of vaccines, including anti-TNF-alpha antibodies (for the treatment of autoimmune disorders) and anti-C1q antibody, currently in phase I trials for both AD and autoimmunity (10, 11).

In contrast to immune tolerization which may be an adaptive mechanism to prevent autoimmune inflammation, allowing the clearance of damaged self-components, immune training may serve the purpose of overriding immune anergy encountered in various pathologies, including sepsis and cancer (12, 13). Indeed, the peripheral and central innate immune cells are engaged in a balancing act as they must be sufficiently trained to effectively defend the host against pathogens and malignancies, while at the same time tolerant enough to accept self-components, food, the fetus and commensal microbes. Disruption of this fine equilibrium may either cause unchecked inflammation or excessive tolerance (12). Iron interferes with this balance by promoting activation and inflammation, a property exploited in oncology to train tumor-associated macrophages (TAMs) in fighting malignant cells (14). On the other hand, iron chelators oppose training, providing therapeutic benefit in AD. Indeed, deferiprone, currently in phase 2 clinical trials, may soon become the first FDA approved iron chelator indicated for AD (15). Along these lines, a recent preclinical study demonstrated that in response to peripherally applied inflammatory stimuli, microglia can undergo either activation (training) or tolerization, the former augmenting, the latter lowering neuroinflammation (13). This is important for AD because this study also found that training increases while tolerization decreases β-amyloid accumulation, suggesting that central tolerance can be promoted by peripheral stimulation, comprising a novel therapeutic strategy (13). Previous AD studies are in line with this model as it was demonstrated that immune trained subjects (positive for TNF-alpha) presented with memory loss, while the tolerized patients (TNF-alpha negative) lacked cognitive deterioration (16). Moreover, trained microglia and astrocytes were found able to undergo transcriptomic alterations, morphing into antigen presenting cells (APCs) that can establish immunologic synapses with infiltrating peripheral lymphocytes, further perpetuating neuroinflammation (17).

Traditionally, immunological memory has been associated with adaptive immunity, however novel studies indicate that innate immune cells, including microglia and astrocytes can “recall” prior vaccinations or infections (13). For example, β-glucan, a cell wall component of Candida Albicans and bacillus Calmette-Guerin (BCG), was shown to train monocytes, improving the outcome of sepsis-induced immune paralysis (18). Indeed, the development of treatments based on innate immune memory manipulation has been encouraged by the recent FDA approval of immune training-inducers, the BCG vaccine for the treatment and prophylaxis of urinary bladder carcinoma *in situ* (CIS) and muramyl tripeptide for osteosarcoma (19). Along these lines, innate immune memory manipulation may improve AD outcome by de-escalating microglia via peripherally-applied tolerizing stimuli (13). Since tolerized immunity, unlike immunosuppression, is antigen specific and natural, harnessing it may not only benefit AD but also cancer and autoimmune disorders (20, 21). The recent finding that altered endogenous molecules, including dysfunctional mitochondria, can trigger microglial training will likely result in novel AD strategies, including mitophagy-activating therapeutics (22).

In this article we look beyond iron chelators, focusing on lowering the consequences of iron pathology, especially microglial training and defective tolerization. The immunotherapeutic agents discussed here include inflammasome inhibitors, histone deacetylase inhibitors, activators of co-inhibitory receptors, acetylcholinesterase inhibitors and selective serotonin reuptake inhibitors.

## Iron Homeostasis and the Innate Immune System

Iron is an indispensable but potentially toxic trace element and for this reason it has to be circulated throughout the body attached to its carrier protein, transferrin (Tf). Dietary iron is absorbed in the gut by crossing the enterocyte brush border membrane via divalent metal-ion transporter 1 (DMT-1). After conversion from ferrous to ferric iron by hephaestin and/or ceruloplasmin it is exported through the basolateral membrane via ferroportin (FPN) channels (23).

Novel studies have demonstrated that gram-negative commensal gut microbes are able to generate more than one type of LPS, thus activating or inhibiting innate immune responses (24). For example, Escherichia coli LPS was found to be an activator of innate immunity, while LPS derived from *Bacteroides dorei* was linked to immune tolerance (25). Interestingly, *Bacteroides fragilis* LPS (BF-LPS) was shown to contribute to autoimmune pathology by selectively inhibiting innate immune tolerization (26). Since the host and microbes share a common iron pool, its availability is directly linked to the survival and growth of the LPS-shedding bacteria.

Iron enters the CNS together with Tf (iron-Tf complex) by binding to transferrin receptor-1 (TFR-1) expressed by the endothelial cells of brain capillaries. After crossing into the cytosol of these cells and satisfying their metabolic needs, excess iron is stored in the cytosolic and mitochondrial ferritin. The remaining iron is exported via FPN into the extracellular space (27).

One of the key roles of the innate immune system is pathogen-mediated intracellular iron sequestration to deny it to bacteria and malignancies which are dependent on this biometal for growth (discussed in The Trained Arm of Innate Immunity section). Iron sequestration is initiated in response to exogenous and endogenous danger signals, consisting of pathogen-associated molecular patterns (PAMPs) or, damage-associated molecular patterns (DAMPs), respectively. For example, LPS, the cell wall component of gram-negative bacteria, is a PAMP that activates microglial TLR-4. In contrast, the endogenously produced ROS is a DAMP that activates the cytosolic inflammasome NOD-like receptor pyrin domain-containing-3 (NLRP3) (28). Iron, itself and/or iron-induced ROS were shown to activate NLRP3 inflammasomes, engendering neuroinflammation (9) (Figure 1).

Iron enters neurons via DMT-1 and TFR-1 receptors but can only exit these cells through FPN, a receptor controlled by hepcidin, the master regulator of iron metabolism. Hepcidin is biosynthesized in the liver, but since only a small fraction of this this peptide hormone crosses the brain-blood barrier (BBB), the brain produces its own hepcidin. High hepcidin levels are associated with intracellular iron accumulation, as this biomolecule binds to FPN, internalizing this receptor (29). LPS and pro-inflammatory cytokines stimulate hepcidin production, inducing hypoferremia, a mechanism involved in the anemia of chronic inflammation pathogenesis (30). Interestingly, astrocytes were shown to induce neuronal death in response to LPS-activated microglia and to synthesize hepcidin via the IL-6/STAT3 pathway, likely linking hepcidin to A1 astrocytes (31). In addition, iron activates nuclear factor kappa-light-chain-enhancer of activated B cells (NF-kB), a transcription factor involved in innate immune training and inflammation (27).

Microglia are yolk-sac-derived myeloid cells, comprising up to 20% of the total brain glial cell population (32). Their main function consists of brain parenchyma surveillance, searching for endogenous or exogenous danger signals to which they respond by activation (33). Another major function of microglia, assisted by astrocytes, consists of tolerization or acceptance of self-molecules, food, commensal microbes, and the fetus. This task is accomplished by phagocytosis via prompt clearance of molecular debris, including dead or dying cells that were shown to trigger inflammation and autoimmunity when allowed to accumulate (34). Taken together, disruption of phagocytosis can lead to unchecked inflammation, impaired immune tolerance and autoantibodies against self-components. Indeed, increased levels of autoantibodies were documented in AD, suggesting that autoimmunity may play a role in this disorder (35).

## The Trained Arm of Innate Immunity

Pathogen-mediated iron sequestration is an effective mechanism that protects the host against bacteria and malignancies at the risk of inflammation and ROS triggered by increased intracellular iron (36). To lower this risk, iron must be properly stored into the cytosolic and mitochondrial ferritin, a process that may be disrupted by inflammation and age-related pathology. In the CNS, pathogen-mediated iron sequestration is initiated upon detection of PAMPs and/or DAMPs by microglia and astrocyte, followed by the release acute phase proteins, including hepcidin and lipocalin-2 (LCN-2), to internalize iron (29, 37).

Aging augments the expression of hepcidin and LCN-2, which along with impaired ferritinophagy, increases the risk of free intracellular iron and prolonged inflammation via NLRP3 inflammasomes activation (16, 38). Both hepcidin and LCN-2 have been associated with neurotoxicity and AD, despite some studies describing them as neuroprotective (39–42). These findings are contradictory in appearance only because hepcidin and LCN-2 may have both beneficial and detrimental effects, depending on the phase of inflammation. For example, during the acute phase, iron sequestration may be adaptive as it is denied to pathogens, limiting infection and inflammation. On the other hand, with chronic inflammation and increasing age, intracellular iron may become detrimental as it activates NLRP3 likely overwhelming immune tolerization and triggering neuronal loss. In addition, iron also activates NF-kB implicated in tolerization disruption and autoimmunity when in excess or inappropriately activated (43) (Figure 1). Iron may impair immune tolerance directly by altering microglial secretome, enhancing the release of TNF-alpha, IL-1, and C1q, cytokines that promote A1 astrocytes (1). Indeed, the pathological phagocytosis that A1 cells engage in may reflect the loss of neuronal and oligodendrocytic tolerization. Interestingly, a different study reported A1 astrocytes' upregulation in healthy elderly individuals, linking these cells to the chronic inflammation of aging or inflammaging, considered by some an AD prodrome (44, 45). Moreover, a novel study found that glucagon-like peptide-1 receptor (GLP1R) agonists can block the conversion of trophic to A1 astrocytes, suggesting that Diabetes mellitus type 2 treatments, including liraglutide, may benefit inflammaging and AD (46). Interestingly, liraglutide was documented to lower inflammatory responses by decreasing the expression of NLRP3, suggesting that this drug may function as an inflammasome inhibitor (47).

**Figure 2:**
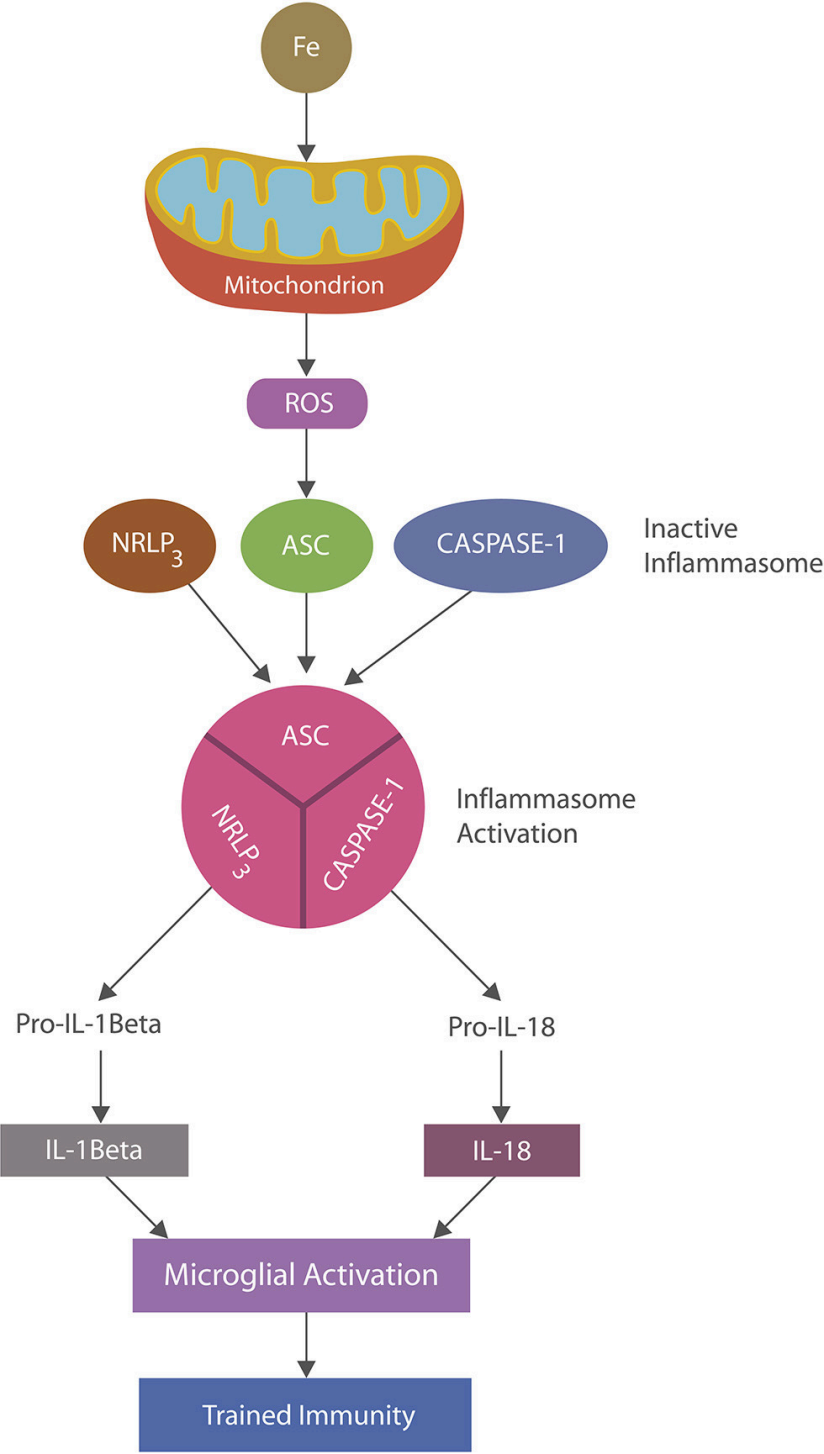
NLRP3 inflammasomes activation: iron-damaged mitochondria, acting as a DAMP, directly or via ROS triggers inflammation by activating NLRP3 inflammasomes. Inflammasomes are comprised of a sensor, the nucleotide-binding oligomerization domain (NOD), in this case the (NOD)-like receptor protein 3 (NLRP3), apoptosis-associated speck-like protein containing a caspase recruitment domain (ASC) and caspase-1 which, upon stimulation, assemble and cleave premature IL-1 beta and IL-18 into their biologically active forms, activating microglia (trained immunity).

Inflammasomes are cytosolic multiprotein complexes activated in innate immune cells in response to PAMPs or DAMPs (16, 48). Inflammation is ignited by the inflammasome assembly with caspase1-induced maturation of interleukin-1β and IL-18 which in turn activate multiple inflammatory pathways (49) (Figure 2). NLRP3 inflammasomes, abundantly expressed by microglia and astrocytes, may play a major role in AD as they can be activated by beta amyloid, iron and ROS, maintaining chronic inflammation (48, 50, 51). Indeed, continuous immune training in inflammaging and AD was associated with perpetually elevated NLRP1 and NLRP3 mRNA levels (52–54). Similarly, chronic NLRP3 activation was associated with atherosclerosis and Western diets, linking metabolism and chronic inflammation (55, 56).

Recently the mitochondrion was identified as the interface connecting immunity and metabolism as this organelle contains the mammalian target of rapamycin (mTOR), a protein involved in shifting the metabolic gears from oxidative phosphorylation (OXPHOS), characterizing tolerized immunity, to aerobic glycolysis, marking immune training (11). Iron has been demonstrated to induce mitochondrial damage, probably lowering OXPHOS and prompting NLRP3 inflammasome assembly and microglial training (57, 58) (Figure 2). In fact, iron-disrupted OXPHOS may leave aerobic glycolysis as the only metabolic option for microglia and a possible source of neuroinflammation in AD. Along these lines, a recent study described a direct cross-talk between mTOR and iron metabolism, suggesting a connection between this biometal and aerobic glycolysis (59, 60). Mitophagy or elimination of damaged mitochondria (including that which is iron-induced) was reported to inhibit NLRP3 and lower immune training in AD models, pointing to mitophagy as a potential AD treatment (61–64). Interestingly, liraglutide, a GLP-1R agonist, was shown to promote mitophagy in addition to, or because of NLRP3 inhibition, indicating a potential “off label” use of this drug (65).

A recent preclinical study linked the NLRP3 component, apoptosis-associated speck-like protein containing a caspase recruitment domain (ASC), with the prion-like seeding of beta amyloid (66). Similarly, in a previous study, ASC was reported as capable of migrating through the extracellular space (67). Another novel study found a link between NLRP3 inflammasomes and prion disease (68). Furthermore, multiple studies over the past decade established a close connection between various forms of prion diseases and autoimmunity, suggesting a common denominator (69, 70). Interestingly, iron was associated with both prion disease and autoimmunity, and chelation was found therapeutic in CNS autoimmune disorders, including MS and experimental autoimmune encephalomyelitis (EAE) (71–74). For example, brain iron dyshomeostasis was demonstrated in prion disease patients, while another study found a higher incidence of autoimmune disorders in individuals with hemochromatosis (71, 74). A preliminary study found that deferiprone, a BBB-crossing iron chelator, was therapeutic in MS patients (73). Moreover, some iron chelators were found beneficial in AD and Parkinson's disease (PD), possibly pointing to the role of autoimmune inflammation in these disorders (75–77). Indeed, over the past decade, several studies have associated autoimmunity with AD as serum and CSF autoantibodies against β-amyloid, tau, ceramide and adenosine triphosphate (ATP) synthase were detected in this disorder (35, 78–82). Interestingly, these molecules were also associated with iron and NLRP3, making it worthwhile to search for ASC autoantibodies in AD (83–87).

## The Tolerized Arm of Innate Immunity

Effective protection from pathogens and malignant cells is an important function of the innate immune system, but an equally important task is protection against autoimmune inflammation by maintaining immunologic tolerance to self-structures, food, the fetus, and commensal bacteria. Tolerized immunity or absence of immune responses to repeated or prolonged LPS stimulation was originally described in rodents which survived the administration of a lethal endotoxin dose after previous exposure to sublethal concentrations (88). The adaptive role of this mechanism in the CNS may include “tolerating” elevated levels of LPS demonstrated in the brains of aging individuals and AD patients (2). Indeed, aging and AD have been associated with increased gram-negative bacteria titers in the GI tract and gums (89). For example, individuals with AD were found to have three times higher LPS blood levels compared to controls (90). Interestingly, novel studies have shown that *B. fragilis* generates a structurally different LPS (BF-LPS), that selectively inhibits immune tolerization, possibly contributing to the pathogenesis of both autoimmune disorders and AD (25, 26).

Aside from the LPS response, brain tolerized immunity may serve the purpose of inhibiting inflammation during phagocytosis of apoptotic cells, synapses, or damaged endogenous molecules, a process we define as tolerant phagocytosis (91, 92). Preclinical studies have shown that disruption of this pathway and accumulation of molecular debris activates autoimmune inflammation, inflicting neuronal damage (93, 94). A novel study has reported that LPS down-regulates the expression of the triggering receptor expressed on myeloid cells 2 (TREM2), the microglial receptor associated with immune tolerance (95) (Figure 1). Apolipoprotein E (ApoE) is a TREM2 ligand and their interaction may be necessary for tolerant phagocytosis (96–98). Indeed, TREM-2 blockade was demonstrated to exacerbate EAE and MS, linking this receptor to the CNS autoimmune disorders (99, 100).

A recent pivotal study established the existence of an ApoE-TREM-2 axis which under normal circumstances engenders tolerized or homeostatic microglial phenotypes (101). In contrast, a dysfunctional ApoE-TREM-2 axis was associated with deficient tolerization and unopposed inflammation. For example, ApoE4 isoform, linked to late onset AD, presents with poor TREM-2 binding, leading to the activation of NLRP3 and immune training instead of tolerization (102, 103).

In AD, the tolerized arm of innate immunity appears to be impaired as persistently elevated LPS brain levels fail to induce tolerance, probably triggering the autoimmune elimination of neurons and oligodendrocytes via A1 astrocytes (1, 2, 98, 104). Moreover, LPS elevation in AD was linked to iron as dormant microbes, including *B. fragilis*, may survive in the CNS in an inactive state limited by the lack of free iron (6). During aging, impaired iron homeostasis provides these microbes with enough metal to resume their growth and LPS shedding, possibly explaining the LPS upregulation observed in AD (105). Indeed, iron may facilitate *B. fragilis* resuscitation in the brain as this microbe was shown to require iron or heme for surviving outside of the GI tract (106). In addition, iron-induced endothelial cells' damage may open the BBB (a property exploited in the treatment of gliomas), allowing microbial passage (107, 108). On the other hand, iron chelators were demonstrated to inhibit *B. fragilis* growth, lowering LPS brain levels, perhaps explaining some of the beneficial effects of these compounds in AD (109). In addition, LPS, not only promotes ferritinophagy, but also the astrocytic expression of hepcidin via IL6/STAT3, further augmenting the cellular free iron pool along with the chances of dormant bacterial resuscitation (31).

**Figure 3:**
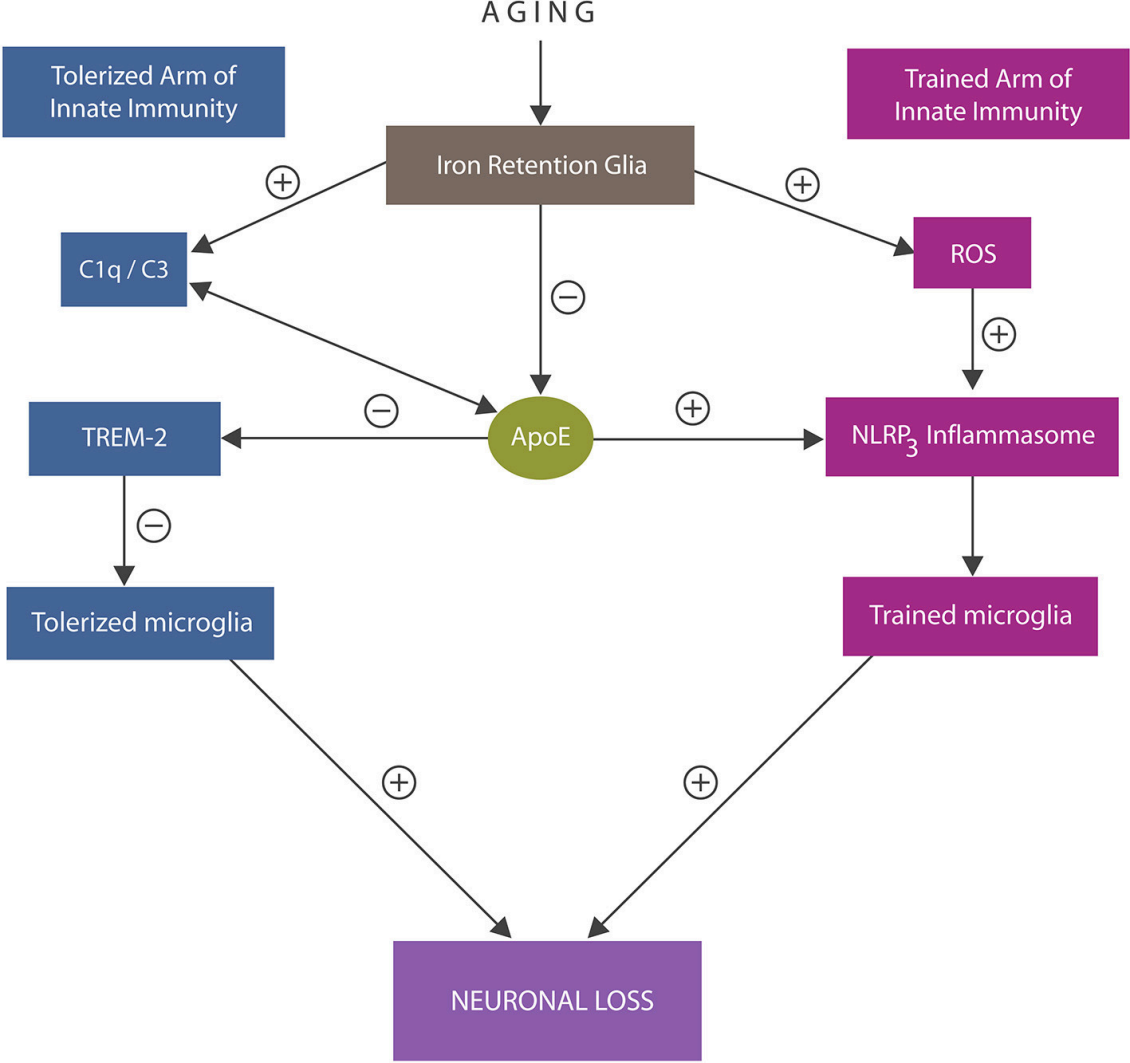
An overview of the trained and tolerized arm of innate immunity. Trained arm: excess iron and ROS trigger activation of NLRP3 inflammasomes with excessively trained microglia and neuroinflammation that may override tolerization. In addition, iron lowers ApoE expression and alters C1q and C3, further impairing tolerization via ApoE -TREM-2 axis. Both excessive immune training or insufficiently tolerized microglia may trigger neuronal loss. ApoE4 isoform binding C1q triggers inappropriate elimination of healthy synapses by astrocytes, likely by promoting the A1 cells.

At the molecular level, tolerized immunity was shown to be associated with microglial TREM-2 receptors, interacting with anti-inflammatory molecules, including ApoE, and complement components C1q/C3 (110–112) (Figure 3). In addition, tolerized immunity was shown to be modulated by iron-dependent hypoxia-inducible factor 1α (HIF-1α), suggesting another tolerization target (113).

Under physiological circumstances C1q and C3 facilitate phagocytosis by tagging dysfunctional synapses for engulfment by microglia and astrocytes (114, 115). In addition, C1q enhances immune tolerance and inhibits NLRP3-induced immune activation (116). Pathologically, deficient C1q was found in over 90% of systemic lupus erythematosus (SLE) patients, linking this protein to autoimmune inflammation (117). In contrast, ApoE4-induced C1q upregulation was associated with inappropriate elimination of healthy synapses by astrocytes, connecting this phenomenon to autoimmunity and the A1 cells (118). Indeed, the development of anti-C1q antibodies may comprise a novel therapeutic strategy for both AD and autoimmunity (10).

Iron disrupts tolerized immunity by lowering ApoE expression in microglia, impairing the ApoE-TREM-2 axis and amyloid plaque phagocytosis (119–121). Indeed, ApoE-immunoreactive microglia were found closely associated with senile plaques, suggesting a key role in AD pathology (122). In addition, iron disrupts tolerized immunity by upregulating C3 and altering the C1q expression, likely preventing their ApoE binding (123, 124). Indeed, C3 upregulation was linked to increased beta amyloid and phosphorylated tau, linking once more iron to AD pathogenesis (110, 125) (Figure 3). Other recent studies connected iron-upregulated C3 with the A1 astrocytes by demonstrating high complement levels in astrocyte-derived exosomes obtained from AD patients (114, 126).

**Figure 4:**
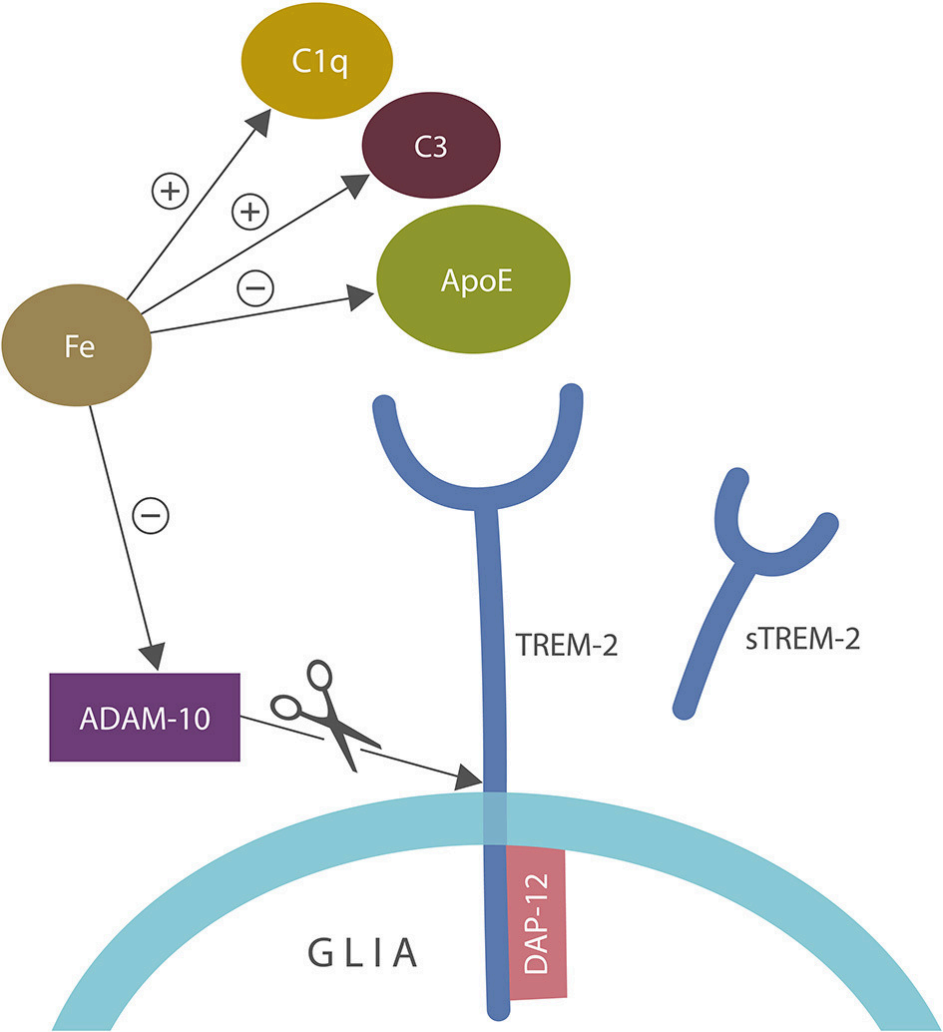
A closer view of microglial ApoE-TREM-2 axis: ApoE, C1q, and C3 engender tolerant phagocytosis (lack of inflammatory response during the clearance of damaged self-components). Iron disrupts this process by promoting prolonged inflammation that overrides immune tolerization. In addition, iron impairs tolerization by altering C1q and ApoE along with C3 upregulation. Iron also inhibits alpha secretase (ADAM-10) which cleaves tolerant TREM-2 receptor into sTREM-2, likely impairing phagocytosis. DAP-12 is a TREM-2 adapter, anchoring this receptor into the cell membrane. ADAM-10 is the same enzyme that cleaves amyloid precursor protein (APP), preventing the formation of amyloid-beta peptide.

Aside from ApoE and C1q/C3, iron inhibits α-secretase or ADAM-10, the enzyme involved in the generation of soluble TREM-2 (sTREM-2), linking again this biometal to autoimmunity (102, 123, 124, 127–129) (Figure 4).

Adenosine-triphosphate (ATP) synthase, the mitochondrial enzyme involved in ATP biosynthesis, is a newly identified AD target, especially because extracellular ATP (eATP) is a potent NLRP3 activator associated with autoimmune disorders (81–85). This is relevant as preclinical studies show iron-associated augmentation of F1 and F0 components of ATP synthase with loss of mitochondrial ATP, possibly suggesting ATP displacement to the extracellular compartment (130). In addition, iron-binding protein, lactoferrin clears eATP, presenting with a beneficial role in AD (see the section on exosomes and Inflammasomes). Furthermore, blockade of purinergic receptors P2Y1 was associated with improved cognition in AD models, while P2X7 inhibition was found beneficial in autoimmune disorders, linking eATP and iron to impaired immune tolerance (87, 130, 131).

## The Innate-Adaptive Continuum

Novel preclinical studies indicate a close cooperation between the innate and adaptive immune systems via NLRP3 inflammasomes and IL-1β (132, 133). Microglia and astrocytes communicate extensively with peripheral lymphocytes, inducing their activation and CNS migration (10, 134). IL-1β is known for increasing BBB permeability, facilitating the infiltration of peripheral cytotoxic T-cells (CTCs) into the CNS (135). In addition, iron is known for altering the BBB by inflicting endothelial cells damage, facilitating peripheral cells access to the CNS (107). In addition, peripheral lymphocytes may access the brain via the newly discovered CNS-draining lymphatics (17).

The newly arrived cells were shown to activate NLRP3, attracting more blood-borne immune cells into the brain (136). On the other hand, tolerized glia may orchestrate the CNS import of T-regs or the newly identified regulatory B1a cells which in turn lower microglial training (137–140). In addition, B-cells have been known to facilitate the recruitment of T-regs to the CNS, augmenting their immunosuppressive activities (141).

T-regs, the preservers of immune tolerance, were found to delay AD progression, linking this condition to autoimmune inflammation (142, 143). The anti-inflammatory IL-10 released by tolerized microglia and T-regs was demonstrated to negatively regulate iron metabolism, possibly explaining the beneficial role of Tregs in AD (144). In addition, IL-4 also regulates iron metabolism and promotes immune tolerance (145). Indeed, iron has been shown to facilitate the CNS infiltration of peripheral CTCs, while iron chelation produces the opposite effect (146). Furthermore, IL-10 was demonstrated to inhibit aerobic glycolysis, the metabolic driver of trained immunity, promoting the tolerant OXPHOS (144, 147, 148). Interestingly, a recent preclinical study discovered a CNS population of cerebral T-regs, documenting that aside from blood borne, the brain maintains its own tolerogenic lymphocyte population (139). Other novel studies demonstrated the existence of various T-regs subpopulations, expressing different co-inhibitory T-cell receptors, including the T-cell immunoglobulin and ITIM Domain (TIGIT) that regulates immunity exclusively via iron-lowering IL-10, linking this receptor to iron downregulation (149, 150).

Co-inhibitory receptors, including cytotoxic T-lymphocyte associated protein 4 (CTLA-4), programmed cell death protein 1 (PD-1), lymphocyte-activation protein 3 (Lag-3), and TIGIT play an essential role in the prevention of autoimmune disorders by promoting tolerized responses (151, 152). A growing body of evidence reveals that dysfunctional co-inhibitory receptors may contribute to AD neuroinflammation as under normal circumstances they function to inhibit inflammation-generating co-stimulatory signals (153). For example, upregulation of PD-1 expressed on both microglia and T-regs, was found beneficial in AD animal models (154). Another study demonstrated that non-PD-1 expressing T-regs were upregulated in mild cognitive impairment (MCI), demonstrating the beneficial role of PD-1 receptors in preventing the development of full-blown AD (155). Indeed, tolerized microglia also express PD-1, activating T-regs and probably facilitating their CNS import (156, 157).

These findings suggest that other co-inhibitory receptors, including TIGIT, may activate T-regs. Interestingly, a recent study found elevated TIGIT in the peripheral blood of healthy elderly individuals (158). As upregulated A1 astrocytes were also found in normal aging, TIGIT and A1 may comprise novel AD risk markers (45). In addition, as TIGIT modulates immunity via the iron-lowering IL-10, this receptor may be elevated in normal aging in a compensatory manner in response to iron overload (149). TIGIT is yet to be demonstrated on glial cells, however like PD-1, it may be a coordinator of adaptive and immune responses.

Taken together, this data suggests that innate and adaptive immunity operate on a continuum as trained immunity may activate CTCs, while tolerized immunity the T-regs, therefore targeting trained and tolerized immunity may lower the CNS infiltration of peripheral lymphocytes.

## Microglial Reprograming: Trained and Tolerized Therapies

The recent FDA approval of immune training-inducers prompted research for similar therapies for cancer and neurodegeneration (18). Immune stimulatory vaccines, comprised of antigen and pro-inflammatory molecules, activate TLRs or NLRP3, inducing immune activation. These therapeutics have been utilized to train APCs and reverse cancer or sepsis-induced T cell anergy. Iron as an inducer of trained immunity has been utilized to activate TAMs and several nanoparticles are currently being designed for its delivery (14). On the other hand, the development of specific tolerized therapies, including “inverse vaccines,” capable of improving pathology without causing generalized immunosuppression, has been vexing (20). The same is true regarding the first-generation AD vaccines, like AN1793, based on anti-Aβ1-42 antibodies which was abandoned due to adverse effects (159). However, despite these draw backs, a new generation of AD vaccines, including anti-C1q mentioned above, are currently being developed. Since neuroinflammation represents the predominant pathology in many neurodegenerative and even psychiatric disorders, de-escalating iron-trained microglia may comprise an effective treatment strategy. Indeed, the anti-inflammatory iron chelator deferiprone is currently in phase 2 trials for AD (15, 160). Deferiprone may have a dual mechanism of action, lowering trained immunity and augmenting tolerization, suggesting that simultaneously targeting both arms of innate immunity may yield better therapeutic outcomes than addressing each one in isolation. Another dual-mechanism-of-action compound, hydroxamic acid, is a promising histone deacetylase-6 inhibitor (HDAC6i) which presents with the added benefit of being an iron chelator (161, 162). Histone deacetylases are enzymes involved in the removal of acetyl groups from histone proteins, altering the chromatin landscape and gene expression. HDACs have been associated with AD pathogenesis, while some HDACis were found to improve cognition (163, 164). In addition, selective HDAC inhibitors were shown to suppress LPS-trained microglia via IL-6 and TNF-α, suggesting a therapeutic potential in AD neuroinflammation (165). Moreover, HDACs 1 and 2 inhibitors are augmenters of microglial β amyloid phagocytosis, linking these compounds to the immuno-metabolic reprograming via OXPHOS augmentation (165, 166).

## Inflammasome Inhibitors

NLRP3 inhibition to lower immune training and the subsequent neuroinflammation is an emerging therapeutic strategy in AD and traumatic brain injury (TBI) (167–169).

Purinergic receptor blockers, ASC inhibitors, diarylsulphonylurea inhibitors, β-sulfonyl nitrile compounds, and caspase-1 blockers are the broad categories of recently developed NLRP3 inhibitors (170, 171). The most studied compound, MCC950 is a diarylsulphonylurea inhibitor, a potent anti-inflammatory drug and a facilitator of β amyloid clearance in AD animal models (172). Another recently developed molecule, OLT1177, is a β-sulfonyl nitrile which inhibits NLRP3 by limiting ROS generation after LPS challenge. This compound is currently in phase I clinical trials.

Suppressing NLRP3 by ASC inhibition is an attractive AD strategy as ASC was recently found to disseminate β amyloid throughout the CNS (66, 172). Interestingly, saxagliptin, a dipeptidyl peptidase-4 (DPP-4) inhibitor, which increases plasma GLP-1 concentration, appears to selectively inhibit ASC, suggesting a role in preventing the spread of AD pathology (173). This is significant since GLP-1R agonists were found to inhibit the conversion of trophic into A1 astrocytes, probably linking ASC to this neurotoxic phenotype (1, 46). Moreover, Tranilast, an anti-allergic drug derived from tryptophan, was also reported to selectively inhibit ASC, suggesting a preventive potential in AD (174).

## Of Exosomes and Inflammasomes

Exosomes are (50–150 nm) extracellular nanovesicles released by most cell types, including neurons and glia, which can carry molecular cargos and trigger functional changes in target cells. Exosomes cross the BBB, facilitating the transfer of inflammation as they can carry a variety of peripheral molecules, including inflammasome-derived proteins (175, 176).

Exosomes, involved in innate immune cells' cross talk, participate in microglial training or tolerization by augmenting or lowering neuroinflammation, depending on their cells of origin (17, 177, 178). Novel studies have found that neuronal exosomes have beneficial effects, while astrocytes-derived exosomes (ADE) are mostly detrimental, inducing neurodegeneration. Neuronal exosomes present with the adaptive function of clearing β amyloid by delivering it to microglia for phagocytosis. For this reason, pharmacological facilitation of neuronal exosomes' synthesis could be developed into an AD therapy (176). For example, designer neuronal exosomes were recently developed and utilized in animal models of Parkinson's disease (170). Interestingly, microglial cells were shown to release exosomes that were previously reported to originate from B cells and dendritic cells, suggesting pro-inflammatory roles (179). CNS autoimmune disorders have been associated with exosomes transporting histocompatibility complex (MHC) class II proteins released by interferon-γ activated microglia (180).

ADE were shown to promote the conversion of trophic to A1 astrocytes by releasing exosomes containing complement components C1q and C3 (126, 181–183). Preventing ADE release may comprise another potential AD strategy. Moreover, a novel study has demonstrated that excessive interstitial ATP can facilitate the generation of A1 astrocytes, suggesting clearance of ATP from the extracellular space (ECS) as a potential AD treatment modality (174). Interestingly, the natural iron chelator, lactoferrin, was found to facilitate the ECS removal of ATP, indicating a novel therapeutic application (184). Indeed, a recent study suggested that engineered lactoferrin-containing exosomes could accomplish this goal (185).

## Histone Deacetylases Inhibitors (HDACi)

Innate immune cells are endowed with an epigenetically regulated memory of previous antigen encounters, suggesting that epigenome manipulation may comprise an efficient reprograming strategy in these cells (17). Acetylation and deacetylation of DNA and histone proteins are the most studied epigenetic mechanisms regulating innate immune cells. Indeed, HDACi were shown to lower the LPS-induced microglial training, promoting tolerization (165). For example, valproic acid, a HDACi utilized in the treatment of bipolar disorder and epilepsy, was shown to downregulate the mammalian target of rapamycin (mTOR), restoring OXPHOS, the driver of immune tolerance (11, 186–188). Furthermore, HDACi were recently shown to augment astrocytic ApoE secretion (189). Since ApoE inhibits NLRP3 activation, HDAC inhibition may have a potential therapeutic value in the fight against AD neuroinflammation (Figure 3).

HDACi are facilitators of ferritin storage, lowering intracellular free iron and the risk of NLRP3 activation (161, 190). In addition, HDACi reverse the iron-induced augmentation of glycogen synthase kinase-3β (GSK3β), a pro-inflammatory enzyme implicated in AD (191, 192). GSK-3β inhibitors, including lithium, were shown to promote immune tolerance, suggesting a therapeutic role in lowering iron levels (193–195). Interestingly, the tolerizing receptor TREM-2 is a negative regulator of GSK-3β, further associating this protein with decreasing iron-induced neuroinflammation (191, 196).

## Co-inhibitory T-cell Receptors in AD

Dysfunctional co-inhibitory receptors may contribute to AD neuroinflammation as under normal circumstances, they block co-stimulatory signals, lowering inflammation (153). For example, stimulation of PD-1 receptors, expressed on both microglia and T-regs, was found beneficial in AD animal models (154). On the other hand, increased number of non-PD-1 expressing T-regs in MCI, shows the importance of this co-inhibitory receptor in AD prevention (155).

T cell activation by APCs is crucial for mounting adequate adaptive immune responses. In the CNS, microglia and astrocytes can become APCs, engendering immunologic synapses with peripheral T cells (10). The proliferation, differentiation, and migration of these cells is triggered by the interplay between co-stimulatory and co-inhibitory signals which fine tune T cell responses (197). For example, the co-stimulatory receptors promote effector T-cells function, while co-inhibitory receptors function to inhibit co-stimulatory signals, promoting T-regs (6).

Activation of co-inhibitory receptors, including CTLA-4, PD-1, LAG-3, TIM-3, and TIGIT, induce immune tolerance, therefore their stimulation would be beneficial in autoimmune disorders (198). On the other hand, their blockade was shown to train TAMs in a manner similar to iron and eliminate cancer cells (199). Interestingly, TIGIT competes with the co-stimulatory signal CD226 for a common ligand CD155, suggesting that promoting the TIGIT-CD155 axis may lower intracellular iron as TIGIT operates exclusively via the iron-lowering IL-10 (200). Indeed, IL-10 or CD-155 loaded exosomes may enhance the co-inhibitory receptor TIGIT, lowering microglial intracellular iron. In addition, the CD226 genetic variant, Gly307Ser, was associated with several autoimmune disorders, suggesting that CD 226 inhibition may facilitate tolerized immunity and benefit the treatment of these conditions as well (201, 202).

## Tolerized Immunity and Acetylcholinesterase Inhibitors

Acetylcholinesterase inhibitors (AChEIs) are cholinergic compounds widely utilized in AD. AChEIs, including donepezil, rivastigmine, and galantamine function by inhibiting cholinesterase, the acetylcholine-degrading enzyme, increasing its synaptic availability in the cholinergic tracts. Some of these drugs may be modulators of trained or tolerized immunity as they oppose iron-induced NLRP3 activation and the inhibition of ADAM-10, the protective α-secretase, in charge of cleaving both TREM-2 and APP (203–205) (Figure 4). In addition, α7- and α9 nicotinic acetylcholine receptor (nAChR) agonists were found to lower autoimmune inflammation, by augmenting tolerization (206, 207). Moreover, activation of α7nAChRs was found to reverse post-surgical cognitive impairment, suggesting that AChEIs-linked cognitive enhancement may be due to their anti-inflammatory actions (208). On the other hand, a recent study demonstrated that iron and ROS can inhibit α7nAChRs signaling, likely disrupting tolerized immunity (209, 210). Interestingly, conjugation of an AChEI, such as galantamine, with the natural iron chelator, lactoferrin, was recently proposed as an AD therapy (211).

## Tolerized Immunity and Selective Serotonin Reuptake Inhibitors (SSRIs)

Serotonin reuptake inhibitors (SSRIs) are widely prescribed antidepressant drugs that block 5-hydroxytryptamine reuptake in the presynaptic neurons of brain serotonergic tracts, augmenting the synaptic availability of this neurotransmitter. However, these compounds were found to also lower trained immune responses. For example, a recent study indicated that the SSRI, fluoxetine reduces the central and peripheral levels of IL-1β by inhibiting NLRP3 (212, 213).

Another group demonstrated the existence of a cross talk between the brain innate immunity and serotonin signaling, possibly linking major depressive disorder with NLRP3 activation (214). Along the same lines, it was recently shown that indoleamine 2,3-dioxygenase-1 (IDO-1), an enzyme associated with tryptophan catabolism, is abundantly expressed by microglia and astrocytes, connecting serotonergic signaling with tolerized immunity (215). IDO-1 induces immune tolerance by processing tryptophan to L-kynurenine, an inhibitor of immune cells' proliferation and maturation (216). IDO-1 is known for orchestrating mother's immune acceptance of the fetus, however some bacteria and cancers were found to exploit this pathway (217, 218). Indeed, an IDO vaccine to block cancer-induced immune tolerance is currently under development (219). Moreover, IDO-1 contributes to the biosynthesis of quinolinic acid (QUIN), a neurotoxic tryptophan catabolite that was linked to AD pathogenesis (220). Interestingly, iron-QUIN complexes were demonstrated in this neurodegenerative disorder, indicating that QUIN toxicity may be iron-related (221, 222). QUIN was demonstrated to activate microglia and astrocytes, resulting in neuronal death, possibly by inducing A1 cells (223–225).

## Conclusions

The healthy immune system must keep trained and tolerized immunity in balance to ensure effective defenses against pathogens and malignancies, while at the same time accepting the self-components, food, the fetus, and commensal microbes. Iron interferes with this process as it triggers a competition between the host and pathogens. Both biological systems have developed mechanisms to capture iron and deny it to the opposing side. The innate immune cells sequestrate iron to restrict its availability to pathogens, but elevated intracellular iron increases the risk of mitochondrial damage, ROS generation, and autoimmune inflammation.

Aging and iron are associated with increased permeability of both GI and brain microvessels, allowing gut microbes, including *B. fragilis*, to migrate into the CNS and shed LPS, disrupting immune tolerization. Manipulation of trained and tolerized immunity, especially via exosomes and nanoparticles loaded with iron chelators could represent future AD strategies (76). Indeed, iron-oxide loaded exosomes for magnetic hyperthermia have been successfully used in oncology (226).

Tolerogenic immunization also known as “inverse vaccines,” can for example, utilize CD-155 loaded exosomes to enhance the co-inhibitory receptor TIGIT and lower AD neuroinflammation (227). In addition, nanoparticles containing NLRP3-inhibitors, HDACi or ASC blockers may soon become parts of the AD armamentarium.

## Author Contributions

All authors listed have made a substantial, direct and intellectual contribution to the work, and approved it for publication.

### Conflict of Interest Statement

The authors declare that the research was conducted in the absence of any commercial or financial relationships that could be construed as a potential conflict of interest.
